# Rupture of Intestines with Peritonitis

**Published:** 1875-10

**Authors:** G. Winfield Zeigler


A Case of Rupture of the Intestines, Followed by Peritonitis, and Death in Thirty Hours.—*By G. Winfield Zeigler, M.D.*—Michael O., æt. 65, married, laborer, was admitted into the surgical ward of the Episcopal Hospital.

The officer who brought him stated that the injury, which was caused by severe kicks upon the scrotum and lower part of the abdomen by a man with whom he was quarreling, occurred two hours before admission. * * *

The post-mortem was made twenty hours after death. Decomposition had already set in. No external marks of violence could be found except the contusion upon the scrotum observed during life. The scrotum was very much swollen.

The abdomen was very tympanitic and much distended. Upon section, diffuse peritonitis was found.

The cavity was filled with a yellow offensive fluid, mixed with fecal matter.

The intestines were in a high state of congestion, and in the lower third of the ileum there were discovered *two ruptures*, the larger one being about one inch, and the smaller one-fifth of an inch, in length.

The parts of the gut immediately surrounding, to the extent of several inches, were in a gangrenous condition. The other abdominal organs were in a normal state.—*Philadelphia Medical Times.*

M. Wurtz has been nominated Professor of Organic Chemistry in the Faculty of Science of Paris. He consequently resigns the office of Dean of the Faculty of Medicine.

